# Transport Turnover Rates for Human OCT2 and MATE1 Expressed in Chinese Hamster Ovary Cells

**DOI:** 10.3390/ijms23031472

**Published:** 2022-01-27

**Authors:** Xiaohong Zhang, Stephen H. Wright

**Affiliations:** Department of Physiology, College of Medicine, University of Arizona, Tucson, AZ 85724, USA; xiaohong@arizona.edu

**Keywords:** transport, transporter turnover rate, organic cation, kidney

## Abstract

MATE1 (multidrug and toxin extruder 1) and OCT2 (organic cation transporter 2) play critical roles in organic cation excretion by the human kidney. The transporter turnover rate (TOR) is relevant to understanding both their transport mechanisms and interpreting the in vitro–in vivo extrapolation (IVIVE) required for physiologically-based pharmacokinetic (PBPK) modeling. Here, we use a quantitative western blot method to determine TORs for MATE1 and OCT2 proteins expressed in CHO cells. MATE1 and OCT2, each with a C-terminal V-5 epitope tag, were cell surface biotinylated and the amount of cell surface MATE1 and OCT2 protein was quantified by western analysis, using standard curves for the V5 epitope. Cell surface MATE1 and OCT2 protein represented 25% and 24%, respectively, of the total expression of these proteins in CHO cells. The number of cell surface transporters was ~55 fmol cm^−2^ for MATE1 and ~510 fmol cm^−2^ for OCT2. Dividing these values into the different J_max_ values for transport of MPP, metformin, and atenolol mediated by MATE1 and OCT2 resulted in calculated TOR values (±SE, *n* = 4) of 84.0 ± 22.0 s^−1^ and 2.9 ± 0.6 s^−1^; metformin, 461.0 ± 121.0 s^−1^ and 12.6 ± 2.4 s^−1^; atenolol, 118.0 ± 31.0 s^−1^, respectively. These values are consistent with the TOR values determined for a variety of exchangers (NHEs), cotransporters (SGLTs, Lac permease), and uniporters (GLUTs, ENTs).

## 1. Introduction

The kidneys play the primary role in clearing the body of ‘organic cations’ (OCs) [1], including about 40% of prescribed drugs [2,3]. Renal OC secretion is a two-step process: the first is dominated by the electrogenic uniporter, organic cation transporter 2 (OCT2), which mediates basolateral OC uptake from the blood into renal proximal tubule (RPT) cells; the second is dominated by the MATE family transporter, MATE1 (MATE2-K expression is ~10% that of MATE1 [4]), which mediates secondary active apical OC exit (via OC/H^+^ exchange) from RPT cells into the tubular filtrate [5]. Both transporters display an extremely broad selectivity [6,7,8,9]. While this makes them clinically important pathways for drug clearance, it also sets the stage for potentially deleterious drug–drug interactions [10,11,12]. Working in concert, these transporters play a pivotal role in defining the pharmacokinetics (PK) of cationic drug elimination, are primary sites of drug–drug interactions (DDIs), and contribute to the nephrotoxicity of selected compounds [13,14]. Their activity is also central to efforts to use physiologically-based pharmacokinetic (PBPK) models to describe drug clearance and predict potential DDIs and cellular toxicity in humans [15].

The application of PBPK involves extrapolating kinetic parameters obtained in vitro to simulate the impact of transport on drug disposition in vivo [16]. Obviously, the accuracy of the resulting models is influenced by the accuracy of the ‘in vitro arm’ of IVIVE. Similarly, extrapolating in vitro observations to the in vivo situation is influenced by the ‘scaling factors’ used to relate data generated in cultured cells to the very different context that exists in the human body. The scaling approach used in most PBPK efforts to model the impact of transport on renal drug clearance employs ‘relative activity factors’ (RAFs), which typically involves fitting the derived model to in vivo profiles. In other words, application of RAFs to PBPK modeling generally permits retrospective, rather than predictive, analyses (e.g., [15,17,18]). Recently, IVIVE has been applied with relative expression factors (REFs) that are derived from the in vivo abundance of the transporter in the tissue of interest (e.g., kidneys), with the in vitro abundance in the cells used to determine the kinetics of transport [4,18]. The kinetics of transport (i.e., values for J_max_ and K_t_) and the abundance of a transporter (the number functionally expressed at the cell surface) are the parameters typically used to derive a fundamental mechanistic characteristic of carrier-mediated transport: the turnover rate (TOR) of the process, i.e., the maximum rate of substrate translocation supported by a carrier under saturating conditions [19]. Knowledge of the TOR for a particular substrate, determined in vitro, can be used to calculate maximal rates of transport in vivo, given knowledge of transporter abundance in the tissue of interest [20].

Accurate assessment of physiologically relevant values for both the kinetics of transport and transporter abundance are challenging, and comparatively few estimates of TOR for transport have been reported. At least one study has, however, reported TOR values for OCT2- and MATE1-mediated transport in cultured cells that stably expressed these processes [21]. However, the values were substantially lower than the TOR values reported for mechanistically similar processes (i.e., uniporters and exchangers), so we elected to reexamine this issue, applying a different methodological approach to the assessment of transport protein (quantitative western blotting of biotinylated cells surface protein vs. LC/MS-MS of total membrane protein), and to the measurement of transport kinetics. For OCT2, the resulting estimates of TOR were modestly larger than the previous estimate. For MATE, however, our estimates of TORs were markedly larger (>1000-fold), reflecting substantial differences in the estimates of transporter expression and rates of transport.

## 2. Results

### 2.1. Kinetics of MATE1 and OCT2-Mediated Transport

The unitary turnover rate (TOR) for a transporter is the number of substrate molecules translocated across the membrane (alternatively, converted to ‘product’) by one transporter (enzyme) per unit time (typically per second; sec^−1^) when exposed to a saturating concentration of substrate (i.e., when all ‘active sites’ are maximally occupied). TOR is usually calculated by dividing the maximal rate of reaction (J_max_ or V_max_) by the number of functional transporters (enzymes). To determine the first of these parameters, we measured the kinetics of transport of several structurally distinct compounds (MPP, metformin, and (for MATE1), atenolol), acknowledging that different substrates can display different maximal rates of transport (e.g., [22]), into CHO cells that stably expressed either MATE1 or OCT2. We should note that the previous study that determined TOR values for MATE1 and OCT2-mediated transport [21], expressed these transporters in HEK 293 cells. However, we have directly compared the kinetics of transport in both CHO and HEK 293 cells (OCT2, [23]; MATE1, unpublished results); the apparent Michaelis constants were not different between the two cells types, and differences in maximal transport were correlated with levels of expression.

**Figure 1:**
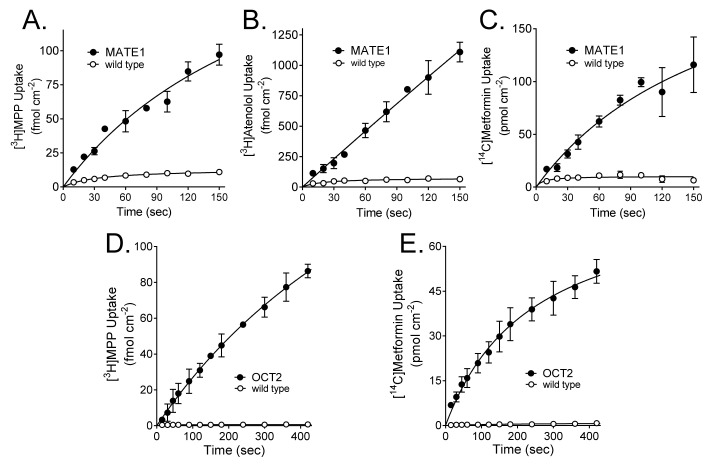
Time courses of uptake of [^3^H]MPP (~10 nM; (**A**,**D**)), [^3^H]atenolol (~0.25 µM; (**B**)), or [^14^C]metformin (~10 µM; (**C**,**E**)) into CHO cells that stably expressed either MATE1 (**A**–**C**) or OCT2 (**D**,**E**). Each point is the average (±SD) of four replicates from a single experiment (each experiment was repeated at least once). Lines describe a hyperbolic rise to a maximum (see text).

Figure 1 shows the time course of net accumulation of MPP, atenolol, and metformin into CHO cells that stably expressed MATE1 (Figure 1A–C; determined in the presence of an outwardly-directed H^+^ gradient), as well as wild type CHO cells. For OCT2, low rates of inhibitable atenolol transport precluded assessment of transport kinetics, so we focused on transport of MPP and metformin (Figure 1 D,E). Net substrate accumulation into cells expressing MATE1 or OCT2 was nearly linear for at least 60 s. Applying an empirical method to estimate the initial rate of influx (using a hyperbolic fit of the time course to estimate the slope at time zero; [24]), the calculated rates of accumulation of these substrates at time zero suggested that the net accumulation at 60 s generally underestimated the true ‘initial’ rate of net accumulation at time zero (uninfluenced by either efflux or unstirred water layers [24]) by approximately 20% for both OCT2 and MATE1. We elected to use 60-s accumulations for subsequent measurements of the kinetics of transport, acknowledging that the resulting values for J_max_ would likely represent a modest underestimate of the ‘true’ value in these cells.

**Figure 2:**
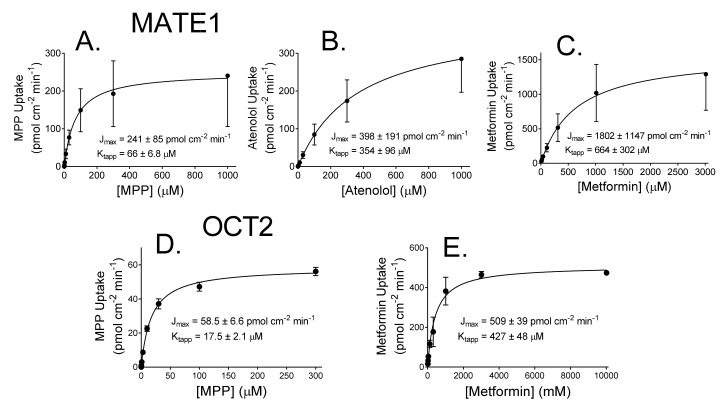
The kinetics of transport for MPP (**A**,**D**), atenolol (**B**), or metformin (**C**,**E**) into CHO cells that stably expressed either MATE1 (**A**–**C**) or OCT2 (D,E). Each point is the average (±SD) of rates determined from 60-s uptakes determined in two or three (OCT2), or four (MATE1) separate experiments. The rates of uptake were corrected for a nonsaturable component of total substrate accumulation (see text), and the lines show the Michaelis–Menten fit of these data (Table 1).

We determined the effect of increasing concentration of MPP, atenolol, or metformin on rates of net accumulation into MATE1-expressing CHO cells (again noting that the MATE1 data reflected the presence of an outwardly-directed H^+^ gradient); and MPP or metformin into OCT2-expressing cells. Net accumulation of these substrates by both transporters was adequately described by the following relationship:

(1) $$\;J=\frac{J_{\max}\left[S\right]}{K_{\mathrm{tapp}}+\left[S\right]}+k_{\mathrm{accum}}\left[S\right]$$

where J is the initial rate of mediated transport from a substrate concentration of [S]; J_max_ is the maximum rate of mediated substrate transport; K_tapp_ is the apparent Michaelis constant, i.e., the substrate concentration in the bulk medium resulting in half maximal mediated transport; and the constant, k_accum_, describes the non-saturable (non-mediated) component of total net substrate accumulation that reflects the combined influence of diffusion, non-specific binding, and incomplete rinsing. As noted previously [24], for polar substrates, such as those used in this study, the primary contributor to k_accum_ is incomplete rinsing of cells following the timed exposure to substrate. Figure 2 shows the kinetic profiles for MATE1- and OCT2-mediated transport of the test substrates (the data presented here were corrected for the non-saturable component of total uptake). From four separate experiments with MATE1, the J_max_ and K_tapp_ for MPP transport were 241 ± 85 (S.D., *n* = 4) pmol cm^−2^ min^−1^ and 66 ± 6.8 µM; for atenolol, 398 ± 191 pmol cm^−2^ min^−1^ and 354 ± 96 µM; and for metformin, 1802 ± 1147 pmol cm^−2^ min^−1^ and 664 ± 302 µM (Table 1). For OCT2, the J_max_ and K_tapp_ for MPP transport (*n* = 3) were 59 ± 6.6 pmol cm^−2^ min^−1^ and 17.5 ± 2.1 µM; and for metformin (*n* = 2), 509 ± 39 pmol cm^−2^ min^−1^ and 427 ± 48 µM (Table 1).

### 2.2. Interaction of the Monoclonal Anti-V5 Antibody with an Epitope ‘Standard’

**Figure 3:**
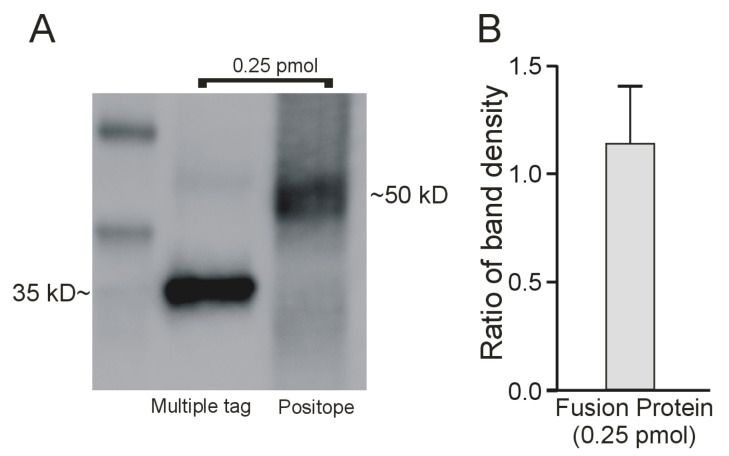
(**A**) Western blot comparing V5 antibody interaction with two different V5 epitope-containing peptides: Multiple tag (Genscript) and Positope (ThermoFisher). (**B**) The ratio of immunoreactive band density (*n* = 3).

**Figure 4:**
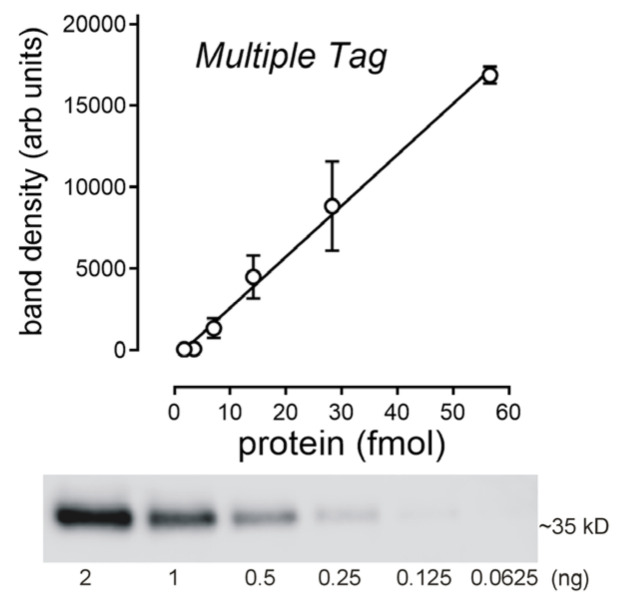
Standard curve relating western blot band density, associated with binding of a V5 monoclonal antibody, to increasing amounts of Multiple Tag protein. Each point is the mean (±SD) of band density determinations determined from blots from four separate experiments. Lower panel shows a representative blot.

To determine the TOR for MATE1- and OCT2-mediated transport, it was necessary to quantify the number of these proteins expressed on the cell surface. Our workflow included quantifying the amount of cell surface biotinylated transport protein as identified in western blots, using the interaction of a V5-antibody with its epitope (which was engineered into both transport proteins at their C-termini). We then compared the intensity of these bands to a standard curve determined in parallel, using a commercial protein that contained the V5-epitope. This approach assumed that the efficiency of interaction of the antibody with its epitope, accessed in a western blot, is independent of its flanking protein sequence(s). Although this is a common assumption, we tested it by comparing the intensity of V5 antibody interaction with two different commercial epitope-tagged fusion proteins, Multiple Tag (Genscript) and Positope (ThermoFisher Scientific), both of which included the V5 epitope sequence (in addition to several other common epitope tags). Western blots revealed a single anti-V5 immunoreactive band for both the Multiple Tag and Positope fusion proteins (35 kD and 53 kD, respectively; Figure 3A). The signals generated by each fusion protein were quantified by densitometry using ImageJ 1.34 s software and showed that the mean ratio of the Multiple Tag to the Positope signal from three experiments was close to 1 (Figure 3B), suggesting that the different flanking fusion proteins minimally affected the efficiency of interaction with the monoclonal anti-V5 antibody. Multiple Tag, however, consistently displayed a more distinct band that did Positope. Therefore, we subsequently used the Multiple Tag fusion protein as the standard for quantitative western blotting. The interaction of the V5 antibody with increasing amounts of Multiple Tag protein, determined through dose-dependent densitometry, was linear over a 32-fold range of sample protein content (Figure 4).

### 2.3. Determination of the Amount of MATE1 and OCT2 in CHO Cell Plasma Membrane

**Figure 5:**
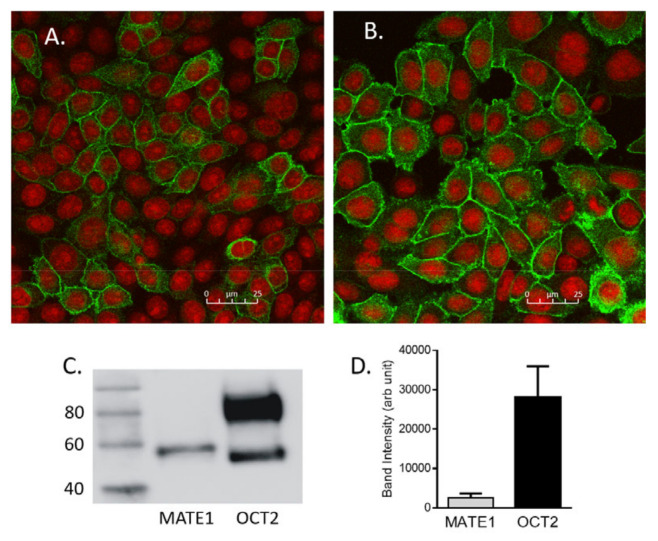
Immunocytochemical localization of (**A**) MATE1 and (**B**) OCT2 in CHO cells that stably expressed these transporters. The transfected constructs both included a C-terminal V5 epitope that was visualized using a fluorescently-tagged (green) secondary antibody. Nuclei were visualized using propidium iodide (red). Both images were linearly stretched, with the stretch to the MATE1 image being equal to that applied to the OCT2 image. (**C**) Western blot comparing the level of total cell (crude membranes) expression of MATE1 and OCT2 in these cells. (**D**) Densitometric assessment of the MATE1 band versus that larger (fully glycosylated) OCT2 band. Bar height reflects the mean (±SD) of band densities measured in three different passages.

Figure 5A shows the immunocytochemical localization of MATE1 (Figure 5A) and OCT2 (Figure 5B) in stably-expressing CHO cells that were stained in parallel and imaged with the same settings; whereas, cytoplasmic reactivity was evident for both proteins, the majority appeared to be found at, or near, the plasma membrane, with substantially larger expression of OCT2, compared to MATE1. Figure 5C shows a western blot of crude (total) membrane prepared from CHO cells that expressed either MATE1 or OCT2. MATE1, which is not glycosylated [5], was found in a single immunoreactive (anti-V5) band of MW ~50 KDa. Immunoreactive OCT2 was found in two bands, the heavier of which is fully glycosylated and reflects mature protein, whereas the smaller is not fully glycosylated and does not traffic to the plasma membrane [25]. The densitometry of the MATE1 band and the larger of the two OCT2 bands suggested that the line of CHO cells that stably expressed OCT2 contains approximately 10-times-more fully mature protein than the MATE1 cell line (Figure 5D).

**Figure 6:**
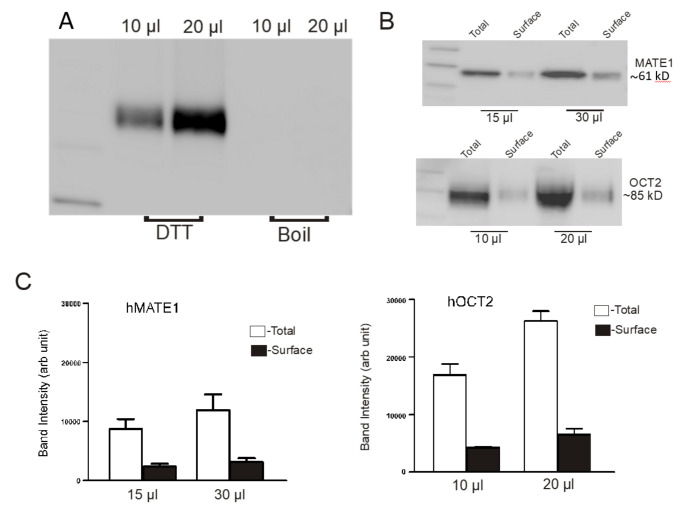
Cell surface expression of MATE1 and OCT2 in CHO cells. CHO cells expressing MATE1 or OCT2 were subjected to the cell surface biotinylation reagent NHSS-biotin, and the biotinylated proteins were recovered from streptavidin-agarose beads. Total protein was obtained by exposing cells expressing MATE1 or OCT2 to lysis buffer for 60 min at room temperature, and total and surface fractions were analyzed using 10% SDS-PAGE. Anti V5 antibody was used to detect the proteins. Bands were quantified using ImageJ software. (**A**) Precipitation of biotinylated MATE1. Lane 1, protein marker, Lane 2 and 3, biotinylated MATE1 was recovered from the beads using an overnight incubation in 50 mM DTT at 4 ^o^C; lane 4 and 5 showed no further release of MATE1 with subsequent boiling of the beads for 5 min at 95 °C, after removing DTT. (**B**) Representative western blots of isolated total protein and surface protein of MATE1 and OCT2. (**C**) Surface and total proteins of MATE1 and OCT2 are expressed as arbitrary units. The height of each bar indicates the mean (±SE, *n* = 3).

The fraction of total mature transport protein expressed at the cell surface was quantitatively determined by comparing total cell MATE1 or OCT2 protein to that expressed at the cell surface, by labelling the latter with the membrane-impermeant biotinylation reagent, NHS-SS-biotin (see Methods). After cell lysis, the biotinylated cell surface proteins were precipitated with streptavidin beads. Figure 6A shows that, after resuspending the biotinylated pellet, an overnight incubation in the resulting solution containing the reducing agent, DTT (50 mM), completely removed biotinylated protein from the beads (no MATE1 protein was detected in samples of the DTT-treated beads that were boiled for 5 min at 100 °C).

Subsequent experiments used DTT to ensure the complete separation of biotinylated proteins from the beads. Representative western blots of isolated total protein and surface protein of MATE1 and OCT2 after cell surface biotinylation are shown in Figure 6B. Figure 6C shows a summary of total protein and surface protein for MATE1 (left panel) and OCT2 (right panel) from three independent experiments. MATE1 expressed at the cell surface was 24.9 ± 1.9% of total MATE1 protein; similarly, cell surface OCT2 protein represented 24.4 ± 2.7% of the total OCT2 protein expressed by the cell.

**Figure 7:**
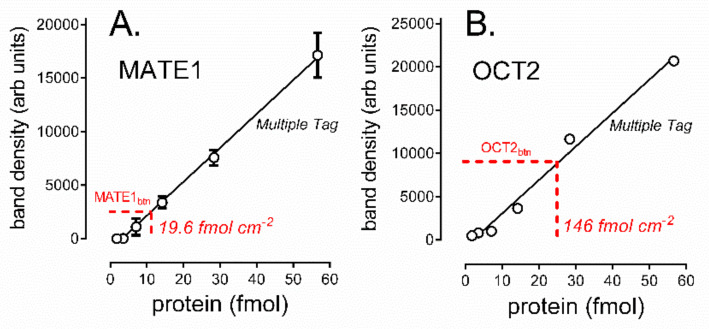
Comparison of western blot band densities for Multiple Tag and biotinylated (**A**) MATE1 protein and (**B**) OCT2 protein. Both plots show data from single, representative experiments. For MATE1 (**A**) Multiple Tag data points (open circles) are the average (±SE) of duplicate measures at each concentration of the standard; for OCT2, these were single band determinations at each standard concentration. Black lines show the linear regression fit to these standards. The horizontal red dashed lines show the biotinylated band densities and calculated levels of expression of transport protein for these experiments.

The amounts of MATE1 and OCT2 protein on the cell surface of CHO cells stably expressing MATE1 and OCT2 transporters were determined by quantitative western analysis with Multiple Tag fusion protein as the V5 expression standard. The densitometric assessments of representative western blots shown in Figure 7 compare the amount of immunoreactive transport protein (expressed in terms of the volume of solution containing total biotinylated cell surface protein obtained from a single well of a 12-well plate) to known amounts of the Multiple Tag standard protein. Figure 7A shows the results from four such experiments, which were used to calculate the amount of transport protein expressed per cm^2^ of confluent cell surface; for MATE1 this was 21.9 ± 18.1 fmol cm^−2^; for OCT2 (*n* = 5) this was 154 ± 70.7 fmol cm^−2^ (Figure 7B; Table 2).

### 2.4. Turnover Rates (TOR) for MATE1 and OCT2

The number of substrate molecules that can be transported per transporter per unit of time (TOR) was determined for the MATE1- and OCT2-mediated transport of MPP, metformin, and (for MATE1) atenolol. The calculation of TOR values involved dividing the maximal rates of substrate transport (J_max_) by the number of cell-surface-expressed transporters (Table 1). The calculated turnover numbers for MATE1 were MPP, 297 ± 201 s^−1^; atenolol 463 ± 313 s^−1^; and metformin, 1894 ± 1282 s^−1^. For OCT2, TOR values were MPP, 8.0 ± 1.4 s^−1^; and metformin, 70 ± 12 (Table 2). For MATE1, whereas the difference in TOR values for MPP and atenolol, and for atenolol and metformin, did not meet the threshold of significance, the difference between MPP and metformin TOR values was significant (one-way ANOVA, *p* = 0.0317). The difference in TOR for OCT2 transport of MPP and metformin was also significant (unpaired t-test, *p* = 0.0003). A two-way ANOVA of the TOR values for MATE1 and OCT2 revealed that the difference in metformin TOR was significant (*p* < 0.05).

## 3. Discussion

The turnover rate (TOR) of a transporter is a primary functional characteristic that, along with the number of transporters expressed in a cell or tissue, defines the maximal rate of transport supported by the process [19]. TORs for any transporter are likely to be substrate-dependent and influenced by the physiological status of the cell in question (e.g., transmembrane substrate gradients, membrane potential, regulatory state). However, knowledge of TOR and the number of functional transporters permits a ‘bottom up’ calculation of transport rates needed for the in vivo-in vitro extrapolation of transport data required for physiologically-based pharmacokinetics (e.g., [16]). TORs for OCT2 and MATE1, both of which are involved in the renal clearance of many cationic drugs [5], were reported previously in a study by Yin et al. [21]. We found those values (~3. 0 and 0.4 sec^−1^ for atenolol transport mediated by OCT2 and MATE1, respectively) rather low, compared to the TORs reported for a number of other transporters (Supplementry Table S1). Consequently, we reexamined this issue, and for both transporters we calculated TOR values that were larger, particularly for MATE-mediated transport (for OCT2, ~10–70 s^−1^; for MATE1, ~300 to 1900 s^−1^) than those previous values. Recalling that determination of TOR for a transporter requires measurement of two parameters (the number of functional transporters expressed by a particular cell or tissue, and the maximal rate of transport mediated by each transporter), we suggest that the discrepancy between these sets of reported values reflects, in large part, methodological differences in determination of both of these parameters.

The qualitative and quantitative assessment of protein expression in cells and tissues increasingly uses LC/MS-MS as the method of choice (e.g., [26,27]), and this was the approach used by Yin et al. in their study of OCT2 and MATE1 [21]. However, a number of alternative approaches have been used to estimate the number of cell-surface-expressed transporters. The most common method, to date, for estimating the number of transporters (for the purpose of calculating TOR), uses ligand binding (Table S1). However, although this has proven effective for some transporters, including the GLUTs (e.g., cytochalasin B; [28]), SGLTs (e.g., phlorizin; [29]), and ENTs (e.g., NBMPR; [30]), this approach requires access to a highly selective, high-affinity ligand (K_d_ in the low nanomolar range or below) to be effective. Other methods used to quantify the number of cell surface transporters include transporter-induced membrane capacitance [31,32], particle counting (from freeze fracture; [33]), and electron paramagnetic resonance [34]. Quantitative western blotting has also been used in many studies (e.g., [35,36,37]), and we elected to apply this method, informed by both previous TOR studies and published recommend practices [38,39], to include validation of standard curves for the epitope under study (in our case, V-5; Figure 4), and restricting our measurements to cell surface (i.e., biotinylated) protein, rather than total cell membrane protein, as commonly employed in LC/MS-MS (e.g., [40]). In intact tissue, cell surface expression of transport protein can range from 60% to less than 10% of total cell expression [41,42]; in our lines of CHO cells that stably expressed MATE1 and OCT2, the cell surface expression was approximately 25% of that in total cell membranes (Figure 6). The amount of cell surface OCT2 protein we determined (~150 fmol cm^−2^, or ~4 fmol µg^−1^ cell surface protein) was about 10% of that reported by Yin et al. (~40 fmol µg^−1^ total membrane protein). However, their use of total membrane protein will have overestimated cell surface OCT2. Assuming cell surface expression was about 25% of total membrane protein, the relevant value for comparison is ~10 fmol µg^−1^, or about twice as much as we determined in our cells. It must also be acknowledged that there is strong evidence that OCT2 in the plasma membrane forms oligomeric complexes through interactions between cysteine residues in the long extracellular loop found between transmembrane helices 1 and 2 [43,44], and it is not clear if such interactions influence transport function. Nevertheless, here we assumed that all transport protein within the plasma membrane is ‘functional’.

The difference in MATE1 expression in our cells and those used by Yin et al. was substantially greater: whereas they reported expression of ~40 fmol µg^−1^ of membrane protein [21], our measured value for cell surface MATE1 was ~0.5 fmol µg^−1^. Even correcting for the likely overestimation of cell surface MATE1 protein obtained using LC/MS-MS, the expression of cell surface MATE1 in our cells appears to have been about 5% that of the cells employed by Yin et al. This comparatively low expression of MATE1 appears to have been real; surface expression of MATE1 was only about 10% of the level of OCT2, evident by both immunocytochemistry (Figure 5A,B) and western blotting (both quantitative, Figure 5D and traditional, Figure 5C). The basis of the low expression was not clear, but certainly the comparatively low value for ‘number of transporters’ contributed to the comparatively large TOR values for MATE1-mediated transport we calculated.

The second parameter involved in calculation of TOR is the maximal rate of transport, i.e., the rate anticipated when the population of cell surface transporters is saturated with substrate. Measurement of accurate transport kinetics is fraught with technical difficulties [45], and methods to improve the accuracy have received attention (e.g., [24,46]). However, as an ‘uptake’ transporter, establishing physiologically-appropriate experimental conditions for the measurement of OCT-mediated kinetics poses comparatively minor problems (as an electrogenic process, the impact of membrane potential on OCT-mediated transport [46] can raise interpretive issues when measuring kinetics in cultured cells [15,47]; however, in the context of the present discussion, the potential error introduced is modest (a factor of 2–3 on J_max_ and/or K_t_)) [15,24,47,48]. Here, we determined the J_max_ of OCT2-mediated MPP and metformin transport (58 and 509 pmol cm^−2^ min^−1^, respectively; Table 1). The larger J_max_ for metformin, compared to MPP, was expected in light of the difference in apparent affinity of OCT2 for the two substrates; high affinity substrates display lower maximal rates of OCT2-mediated transport [22], and was similar to the J_max_ for OCT2-mediated metformin transport reported by Yin and colleagues in a separate study [49]. Our maximal rate values led to calculated TORs of 8 and 70 s^−1^ for MPP and metformin, respectively. The TOR for atenolol transport determined by Yin et al. was about 4 s^−1^ [21], similar to our TOR of 8 s^−1^ for MPP, which in large part reflected comparatively similar values for both J_max_ (~13 vs. 58 pmol cm^−2^ min^−1^ for atenolol and MPP, respectively), and (as noted earlier) levels of OCT2 protein expression.

The same cannot be said for MATE1. Whereas Yin et al. reported a TOR of 0.4 s^−1^ for MATE1-mediated atenolol transport [21], our calculated TOR for the same substrate was over 1000-times larger (462 s^−1^; Table 2), not unlike our TOR values for MATE1-mediated MPP and metformin transport (~300 s^−1^ and ~1900 s^−1^). The difference reflects both the lower number of MATE1 transporters we identified on the cell surface, as discussed previously, and the much higher rates of transport measured in our cells (J_max_ for atenolol of 388 vs. ~13 pmol cm^−2^ min^−1^; Table 1), despite lower levels of membrane expression. We suggest that the higher rates of MATE1-mediated transport reflect the different experimental conditions used in studies with MATE1.

Whereas OCT2 mediates substrate uptake (from the blood), under physiological conditions MATE1 mediates substrate efflux (into the tubular filtrate). Nevertheless, virtually all knowledge of the kinetic and selectivity characteristics of MATEs comes from studies of substrate uptake. The emphasis on assessing uptake (rather than efflux) reflects the comparative ease of measuring the former compared to the latter [50,51], and although the kinetic characteristics of efflux need not be the same as those for uptake [19], it is the usual working assumption (with appropriate interpretive caveats) and the one we used here. However, a second complicating factor that MATEs present reflects the pivotal role of H^+^ concentration on MATE activity, and methodological differences between our study and that of Yin et al. [21] certainly contributed to the higher rates of transport determined in our experiments. The *‘cis’* concentration of H^+^ (in ‘uptake’ studies, this is extracellular pH) acts as a competitive inhibitor of OC transport [51,52]. Consequently, apparent K_t_/IC_50_ values of MATEs for their substrates/inhibitors can be several times lower [51,53] when measured at the elevated extracellular pH values (pH 8 to 8.5) that are frequently used to increase measured rates of transport (e.g., [21,49,53,54]); this was the approach used by Yin et al. [21]. However, MATEs are ‘obligatory’ exchangers [55], and transporter turnover requires that a ‘substrate’ molecule be translocated (probably sequentially) in each direction. This explains why reducing extracellular [H^+^] stimulates MATE-mediated transport (rather than the influence of the outwardly-directed H^+^ ‘gradient’ this condition nominally represents). Nevertheless, it is the absolute concentration of H^+^ at the *trans* face of the transporter that influences how rapidly the transporter can turnover [51]. The stimulation of MATE-mediated uptake observed following acidification of the cytoplasm [56,57] reflects an increase in J_max_, because of the increased availability of exchangeable substrate (i.e., *trans* H^+^; [52]). This is physiologically relevant as it generally results in accelerating the efflux of cytoplasmic OC through exchange for luminal H^+^ [58]. The cytoplasmic pH of RPT cells is in the order of 7.2, whereas that of the filtrate is probably in the order of pH 6.8 [59], resulting in an inwardly directed (~2.5-fold) proton gradient. To mimic this H^+^-driven mode of MATE activity, uptake is often measured following acidification of the cytoplasm, by pre-exposing cells to ammonium chloride, then removing the ammonium (the ‘ammonia pulse’ protocol; [13,60]); this is the approach we used. The comparatively large J_max_ for MATE1-mediated atenolol transport in our study, compared to that reported by Yin et al. [21] (J_max_ of 388 vs. ~13 pmol cm^−2^ min^−1^), was certainly influenced by the acidification of the cytoplasm achieved by our ammonia pulse procedure s (cytoplasmic pH of approximately 6.0; [61]). Indeed, it is likely that the cytoplasmic face of the transporter was effectively saturated with H^+^ [51], thus maximizing turnover of the transporter back to its outwardly-directed conformation and, thereby, producing the largest obtainable J_max_ for uptake of cationic substrates. In contrast, the outwardly-directed H^+^ gradient presumed to be present when measuring MATE-mediated transport at an extracellular pH of 8.0 involves cytoplasmic conditions that will produce much lower rates of OC uptake than those we measured; this certainly contributed to the larger TOR values we calculated.

**Figure 8:**
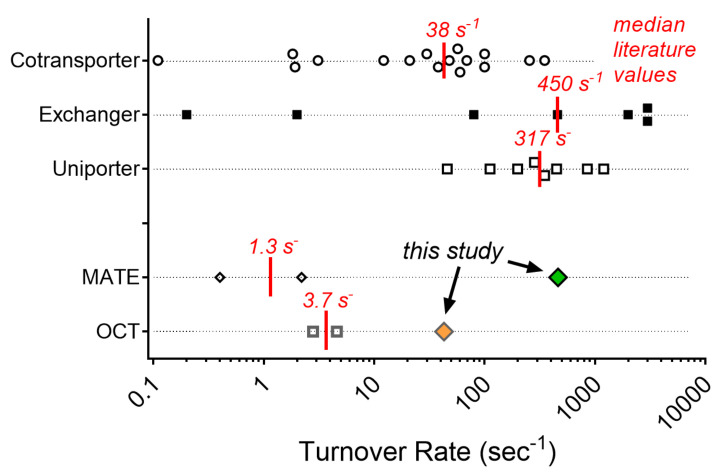
TOR values for selected members of the solute carrier group of transporters (refer to Sup Table 1 for precise TOR values and associated literature references). Vertical red lines show the median values for each mechanistic category of transporter. Values for MATE and OCT transporters reflect those reported in Yin et al. [21] and in the present study.

The TOR values we obtained for OCT2 and MATE1 are larger than those reported by Yin et al. [21]. Although the previous discussion accounted for the basis of some this discrepancy, it worth comparing them to values reported for other transport processes. Though not an exhaustive list, Supplementary Table S1 presents TOR values for a number of processes that represent several different mechanistic classes of transporter, i.e., facilitated diffusion (uniporters), and secondary active co- and counter-transporters (exchangers). Figure 8 displays these values, to facilitate their comparison. OCT2 is a uniporter and a member of the major facilitator superfamily (MFS). It is, perhaps, fortuitous that TOR values have been determined for two families of MFS uniporters, the GLUTs and the ENTs; of the eight TOR values reported, the range of values is from 46 to 1200 s^−1^, with a median value of 317 s^−1^. This range of values is substantially larger than the value of 3.7 s^−1^ obtained by Yin et al. [21], but reasonably similar to the values we obtained, which averaged ~40 s^−1^. MATE1 is an exchanger (counter transporter), and TOR values have been reported for two families of counter-transporters, the Na/H exchangers (NHEs) and the (synaptic) vesicle monoamine transporters (VMATs/SVATs). The MATEs display a structural organization [62] quite distinct from either the VMATs (which are members of the MFS), and the NHEs [63]. The TOR reported for MATE1-mediated atenolol transport was 0.4 s^−1^, whereas the TOR we determined for atenolol was 460 s^−1^. The range of literature TOR values for counter-transporters was 0.2 to 3000 s^−1^, with a median value of 450 s^−1^ (Supplementry Table S1; Figure 8). It is difficult to draw any conclusions from these comparisons; the range of values is just too large. However, we suggest that a TOR of 0.4 s^−1^, in light of the conditions employed in the measurements of transport, certainly represents a significant underestimate of the likely turnover rate of the process under physiological conditions.

PBPK modeling of the impact of transport on drug clearance requires the extrapolation of transport rates determined in vivo, to those expected to occur in vitro. To date, most studies applying PBPK methods to transporters have used ‘relative activity factors’ (RAFs) to scale in vitro rates to rates occurring in vivo that could account for observed pharmacokinetic data; in other words, a retrospective approach (e.g., [15]). There is, however, an increasing trend toward applying ‘relative expression factors’ (REFs) to IVIVE. The REF approach uses information on the in vitro and in vivo abundance of the transporter in the cells used to determine the drug’s in vitro transport and in the tissue of interest (e.g., kidneys) [18]. This approach, in effect, reflects assessment of in vitro TOR values for use in estimating in vivo transport rates, once levels of expression (number of transporters) are known. The quantitative western blot approach we used requires access to a sensitive and specific antibody to the transporters under study. For in vitro work, this requirement was well met by applying an addition of the V5 epitope tag to OCT2 and MATE1, and we suggest that our results confirm the utility of western blotting to make the required measurements of transporter abundance for determining TOR values. This approach, however, cannot be used for in vivo samples. Consequently, LC/MS-MS likely remains the most practical approach for comparison of transporter abundance in in vitro and in vivo samples.

In summary, we determined the turnover rates for OCT2- and MATE1-mediated transport of several structurally distinct substrates and compared these values to those reported in a previous study of these processes [21]. Overall, the TOR values we estimated were larger than those reported previously. For OCT2, the differences in TOR values were modest and, in large part appeared to reflect an overestimation of cell surface expression of transporters. The differences in calculated TOR values for MATE1 were much larger (>1000-fold) and appeared to reflect, in equal parts, a lower level of cell surface MATE1 expression and higher rates of MATE1-mediated transport in our study. It is unclear the extent to which methodological differences in the assessment of transporter expression (i.e., our use of quantitative western blotting vs. application of an LC/MS-MS method in the previous study; [21]) contributed to the observed discrepancy. However, the higher rates of MATE1-mediated transport we determined clearly reflected the experimental protocol used to measure transport, and underscored the importance of determining rates of transport under conditions that most closely reflect the anticipated in vivo situation.

## 4. Materials and Methods

### 4.1. Chemicals

[^3^H]1-Methyl-4-phenylpyridinium (MPP) (specific activity (S.A.), 80 Ci/mmol) was purchased from Perkin-Elmer (Waltham, MA, USA); [^3^H]atenolol (S.A. 3.5 Ci/mmol) and [^14^C]metformin (S.A. 114 mCi/mmol) were purchased from Moravek Biochemicals (Brea, CA, USA). Unlabeled atenolol and metformin were purchased from Sigma-Aldrich (St. Louis, MO, USA) and AK Scientific (Union City, CA, USA), respectively; unlabeled MPP (purity > 99.5%) was synthesized by the Department of Chemistry and Biochemistry, University of Arizona. Ham’s F12 Kaighn’s modified medium was obtained from Sigma-Aldrich (Burlington, MA, USA). Other reagents were of analytical grade and obtained from standard sources.

### 4.2. Cell Culture

Chinese hamster ovary (CHO) cells with a single integrated Flp-In recombination site were obtained from Invitrogen (Carlsbad, CA, USA). Preparation of cell lines that stably expressed hMATE1 (NM_018242.3) and hOCT2 (BC039899.1) (described in [64,65]) used the pcDNA5/FRT/V5-His TOPO mammalian expression vector, which added a 23 amino acid extension to the C-terminus of these proteins that included the V5 (GKPIPNPLLGLDST) and 6xHis epitope tags. Cells were passaged every 3–4 days and maintained at 37 °C in a humidified environment with 5% CO_2_. Continued expression of MATE1 and OCT2 in these cell lines was maintained using hygromycin (200 µg/mL; Invitrogen, Carlsbad, CA) selection pressure; continued expression of the Flp-In recombination site in wild-type (WT; non-transporter expressing) CHO cells was maintained using Zeocin (100 µg/mL; Invitrogen) selection pressure. When seeded into 96-well plates (Greiner; VWR Intl., Arlington Heights, IL, USA) for transport assays, cells were grown to confluence in antibiotic-free medium.

### 4.3. Transport Experiments

Cells were seeded in 96 well plates with 200 µL of cell media containing 550,000 cells/mL or 275,000 cells/mL, and experiments were performed 24 or 48 h later, respectively. To begin an experiment, media was aspirated and the wells were washed for three cycles over 12 s with 300 µL of room temperature (~23 °C) Waymouth Buffer (WB; 135 mM NaCl, 13 mM HEPES, 2.5 mM CaCl_2_·2H_2_O, 1.2 mM MgCl_2_, 0.8 mM MgSO_4_·7H_2_O, 5 mM KCl, and 28 mM D-glucose; pH 7.4) using an automatic microplate washer (Model 405, BioTek, Winooski, VT, USA). For studies of OCT2-mediated transport, uptake was initiated by addition of 60 µL of WB containing radiolabeled substrate and other compounds, as needed. For studies of MATE1-mediated transport, the cells (MATE1-expressing and control cells) were exposed to an ammonia pulse method that we previously used [9] to elevate cytoplasmic [H^+^] to a level (~6.0 pH; [61]) that effectively saturates the cytoplasmic face of the exchanger with H^+^ [51]. Briefly, cells were preincubated for 20 min (room temp) in buffer containing 20 mM NH_4_Cl (the first step in establishing an outwardly-directed H^+^ gradient; [60]). Plates were then placed in the automatic fluid aspirator/dispenser and rinsed/aspirated three times with room temperature WB (pH 7.4), and transport was initiated by aspirating this medium and replacing it with 60 µL of an NH_4_Cl-free medium containing labeled substrate, thereby, establishing a *trans* H^+^ gradient, qualitatively similar to the conditions that support MATE1-mediated transport in renal proximal tubules. For all experiments, transport buffers were added using a VIAFLO 96-well multichannel pipet (Integra Biosciences, Hudson, NH, USA). At specified time intervals, transport was terminated by rinsing with three cycles of cold WB (300 µL). Following rinsing and final aspiration, 200 µL of scintillation cocktail (Microscint 20, Perkin-Elmer, Waltham, MA, USA) was added to each well. The plates were sealed (Topseal-A, Perkin Elmer), and after sitting for at least two hours, accumulated radioactivity was determined in a six-channel multiwell scintillation counter (Wallac Trilux 1450 Microbeta, Perkin-Elmer).

### 4.4. Immunocytochemistry

ICC was generally performed on a confluent monolayer (on a glass cover slip) 24 h after plating. The cells were fixed in ice-cold 100% methanol for 20 min, washed (3×) with PBS (137 mM NaCl; 2.7 mM KCl; 8.0 mM Na_2_HPO_4_; and 1.5 mM KH_2_PO_4_, pH 7), and then incubated for 1 h with a mouse monoclonal anti-V5 antibody (Invitrogen) diluted 1:500 in PBS. The cells were then incubated for 1 h in the dark with fluorescein isothiocyanate-conjugated goat anti-mouse antibody (Molecular Probes, Inc., Waltham, MA, USA) diluted 1:1000 in PBS. To visualize the nuclei, the cells were treated with propidium iodide (5 µg/mL) for 10 min. The cells were washed again, and the coverslips were mounted onto microscope slides using Dako fluorescence mounting medium (Dako Corporation, Carpenteria, CA, USA). A confocal microscope (Nikon PCM 2000 scan head fitted to a Nikon E800 microscope) was used for detection of immunoreactive protein in CHO cells.

### 4.5. Cell Surface Biotinylation of MATE1 and OCT2

The method described here is a minor modification of that described by Pelis et al. [25]. All of the solutions were kept ice-cold throughout the procedure, and long incubations were conducted on ice with gentle shaking. Cells plated to confluence in a 12-well plate were initially washed three times with 2 mL of PBS solution containing calcium and magnesium (PBS/CM; 137 mM NaCl, 2.7 mM KCl, 8 mM Na_2_HPO_4_, 1.5 mM KH_2_PO_4_, 0.1 mM CaCl_2_, and 1 mM MgCl_2_, pH 8.0 with NaOH). The cells were then exposed for 20 min to 0.5 mg/mL sulfo-NHS-SS-biotin (ThermoFisher, Walthan, MA, USA), diluted in PBS/CM (a time and concentration shown previously to result in steady-state cell surface labelling; [25,64]). After biotinylation, the cells were rinsed three times briefly with 3 mL of PBS/CM (the NHS-biotin rinse also contained 100 mM glycine). The cells were lysed on ice for 1 h, with gentle shaking in 1 mL of lysis buffer (150 mM NaCl, 10 mM Tris-HCl, 1% Triton X-100, 1% sodium deoxycholate, and 0.1% SDS, pH 7.4) containing protease inhibitors (200 µM 4-(2-aminoethyl)-bezenesulfonyl-fluoride, 0.16 µM aprotinin, 4 µM leupeptin, 8 µM bestatin, 3 µM pepstatin A, 2.8 µM E-64; Sigma-Aldrich), after which 50 µL of streptavidin-agarose beads (ThermoFisher) were added to the lysates and incubated overnight at 4 °C with constant mixing. After extensive washing with the above lysis buffer, 50 µL of Laemmli sample buffer was added. Proteins conjugated to NHS-SS-biotin were released from the beads by an overnight incubation in 50 mM DTT at 4 °C.

For collection of total cell protein, cells plated to confluence in a 12-well plate were rinsed three times with 2 mL of PBS, removed by scrapping, and centrifuged for 5 min at 1000 rpm at 4 °C. The cell pellet was lysed with 300 µL of lysis buffer containing protease inhibitors (1:250), on ice, with gentle shaking and then centrifuged for 30 min at 14,000 rpm at 4 °C. Fifty µL of Laemmli sample buffer was added to the ‘crude cell membrane’ pellet. Proteins in the sample were separated on 10% SDS-PAGE gels, transferred to PVDF membranes, and immunoreactivity corresponding to the V5-tagged MATE1 or OCT2 construct was visualized, as previously described [25]. Some gels also contained serial dilutions of a commercial GST fusion protein (Genscript, Piscataway NJ, USA; ThermoFisher) that contained the V5 epitope. This material was reconstituted in distilled water, quantified via the bicinchoninic protein assay, after which bovine serum albumin, BSA, was added, to a final concentration of 10 ng.

Densitometry was used to quantify the amount of MATE1 and OCT2 protein expressed in the plasma membrane of CHO cells stably expressing these transporters. Immunoreactive intensity of individual bands was determined from scanned images using ImageJ 1.34s (National Institutes of Health, Bethesda, MD, USA). To obtain the level of immunoreactivity, a band on a western blot was selected, and ImageJ scanned the selected band, averaging the 16-bit gray scale values that were located on each horizontal line. The dimensions of the selection area were set to encompass the largest band on an individual blot, and the selection area was typically kept the same for each subsequent band analyzed. The average 16-bit gray scale values on each horizontal line were summed to obtain cumulative 16-bit gray scale values for each band. The average background gray scale value for each lane was subtracted from each horizontal line average to standardize for differences in background between western blots.

### 4.6. Statistics

Transport data are typically expressed as means ± SD, and generally based on the number of separate experiments conducted on cells at a different passage number. Statistical comparisons were performed using an unpaired *t*-test.

## Figures and Tables

**Table 1:** Kinetics parameters for MATE1- or OCT2-mediated transport of MPP, atenolol, or metformin. Values are means (±SD) for two to four separate experiments. (ND, not determined).

Substrate	MATE1		OCT2	
	J_max_ (pmol cm^−2^ min^−1^)	K_t_ (µM)	J_max_ (pmol cm^−2^ min^−1^)	K_t_ (µM)
MPP	241 ± 84.9 (*n* = 4)	66.0 ± 6.8	58.5 ± 6.6 (*n* = 3)	17.5 ± 2.1
Atenolol	398 ± 191 (*n* = 4)	354 ± 95.9	ND	ND
Metformin	1802 ± 1147 (*n* = 4)	664 ± 302	509 ± 39.0 (*n* = 2)	427 ± 48.0

**Table 2:** Abundance of surface biotinylated transport protein and calculated turnover rates (TORs) for MATE1 and OCT2 stably expressed in CHO cells. TOR values calculated from transport abundance and maximal rates of transport (Table 1).

Substrate	MATE1		OCT2	
	Abundance (fmol cm^−2^)	TOR (s^−1^)	Abundance (fmol cm^−2^)	TOR (s^−1^)
	21.9 ± 18.1		154 ± 70.7	
MPP		297 ± 201		7.1 ± 2.4
Atenolol		463 ± 313		ND
Metformin		1894 ± 1282		62.2 ± 20.5

## Data Availability

Not applicable.

## Supplementary Materials

The following are available online at www.mdpi.com/article/10.3390/ijms23031472/s1.
