# Supplementary material for: Transport Turnover Rates for Human OCT2 and MATE1 Expressed in Chinese Hamster Ovary Cells
Source: Int J Mol Sci. 2022 Jan 27;23(3):1472. doi: 10.3390/ijms23031472 (PMC8836179; doi:10.3390/ijms23031472)
Supplement: Supplementary file 1 [file ijms-23-01472-s001.zip › ijms-1532560-supplementary.pdf]

Table S1. List of Transporter Turnover Rates (TORs) for Representative Transport Processes.

Category	Transporter	TOR (sec^{-1})	Reference
uniport	OCT2	43	current study
	OCT2	2.8	[1]
	OCT1	4.6	[1]
	Hxt7	200	[2]
	GLUT4	283	[3]
	GLUT3	853	[4]
	GLUT4	350	[5]
	GLUT1	1200	[6]
	GLUT1	450	[3]
	ENT1	112	[7]
	ENT1	46	[8]
countertransport	MATE1	463	current study
	MATE1	0.4	[1]
	MATE2-K	2.2	[1]
	VMAT1	0.2	[9]
	VMAT1	0.2	[10]
	SVAT	2	
	NHE1-3	459	[11]
	NHE1-3	80	[11]
	NHE1	3000	[12]
	NHE1	3000	[13]
	NHE	2000	[14]
cotransport	SGLT1	38	[15]
	SGLT1	57	[16]
	rSGLT1	30	[17]
	rbPEPT1	100	[18]
	pSGLT2	60	[19]
	SERT	1.83	[20]
	SERT	3.1	[21]
	NKCC	255	[22]
	NET	0.11	[20]
	MCT1	12.2	[23]
	LacY	69	[24]
	LacY	48	[25]
	DAT	1.92	[20]
	AAP1/NAT2	350	[26]

References

1. Yin, J.; Duan, H.; Shirasaka, Y.; Prasad, B.; Wang, J., Atenolol renal secretion is mediated by human Organic Cation Transporter 2 and Multidrug and Toxin Extrusion proteins. *Drug Metab. Dispos.* **2015,** 43, (12), 1872-81.
2. Ye, L.; Berden, J. A.; van Dam, K.; Kruckeberg, A. L., Expression and activity of the Hxt7 high-affinity hexose transporter of Saccharomyces cerevisiae. *Yeast* **2001,** 18, (13), 1257-67.
3. Nishimura, H.; Pallardo, F. V.; Seidner, G. A.; Vannucci, S.; Simpson, I. A.; Birnbaum, M. J., Kinetics of GLUT1 and GLUT4 glucose transporters expressed in Xenopus oocytes. *J. Biol. Chem.* **1993,** 268, (12), 8514-20.
4. Maher, F.; Davies-Hill, T. M.; Simpson, I. A., Substrate specificity and kinetic parameters of GLUT3 in rat cerebellar granule neurons. *Biochem. J.* **1996,** 315 (Pt 3), 827-31.
5. Goodyear, L. J.; Hirshman, M. F.; Valyou, P. M.; Horton, E. S., Glucose transporter number, function, and subcellular distribution in rat skeletal muscle after exercise training. *Diabetes* **1992,** 41, (9), 1091-9.
6. Palfreyman, R. W.; Clark, A. E.; Denton, R. M.; Holman, G. D.; Kozka, I. J., Kinetic resolution of the separate GLUT1 and GLUT4 glucose transport activities in 3T3-L1 cells. *Biochem. J.* **1992,** 284 (Pt 1), 275-82.
7. Doherty, A. J.; Jarvis, S. M., Na(+)-dependent and -independent uridine uptake in an established renal epithelial cell line, OK, from the opossum kidney. *Biochim. Biophys. Acta* **1993,** 1147, (2), 214-22.
8. Ward, J. L.; Sherali, A.; Mo, Z. P.; Tse, C. M., Kinetic and pharmacological properties of cloned human equilibrative nucleoside transporters, ENT1 and ENT2, stably expressed in nucleoside transporter-deficient PK15 cells. Ent2 exhibits a low affinity for guanosine and cytidine but a high affinity for inosine. *J.Biol Chem.* **2000,** 275, (12), 8375-8381.
9. Gasnier, B.; Krejci, E.; Botton, D.; Massoulie, J.; Henry, J. P., Expression of a bovine vesicular monoamine transporter in COS cells. *FEBS Lett.* **1994,** 342, (3), 225-9.
10. Peter, D.; Jimenez, J.; Liu, Y.; Kim, J.; Edwards, R. H., The chromaffin granule and synaptic vesicle amine transporters differ in substrate recognition and sensitivity to inhibitors. *J. Biol. Chem.* **1994,** 269, (10), 7231-7.
11. Cavet, M. E.; Akhter, S.; de Medina, F. S.; Donowitz, M.; Tse, C. M., Na^+/H^+ exchangers (NHE1-3) have similar turnover numbers but different percentages on the cell surface. *Am. J. Physiol.* **1999,** 277, (6 Pt 1), C1111-21.
12. Siczkowski, M.; Davies, J. E.; Ng, L. L., Activity and density of the Na+/H+ antiporter in normal and transformed human lymphocytes and fibroblasts. *Am. J. Physiol.* **1994,** 267, (3 Pt 1), C745-52.
13. Siczkowski, M.; Ng, L. L., Culture density and the activity, abundance and phosphorylation of the Na+/H+ exchanger isoform 1 in human fibroblasts. *Biochem. Biophys. Res. Commun.* **1995,** 209, (1), 191-7.
14. Dixon, S. J.; Cohen, S.; Cragoe, E. J., Jr.; Grinstein, S., Estimation of the number and turnover rate of Na+/H+ exchangers in lymphocytes. Effect of phorbol ester and osmotic shrinking. *J. Biol. Chem.* **1987,** 262, (8), 3626-32.
15. Hirsch, J. R.; Loo, D. D.; Wright, E. M., Regulation of Na+/glucose cotransporter expression by protein kinases in Xenopus laevis oocytes. *J. Biol. Chem.* **1996,** 271, (25), 14740-6.
16. Loo, D. D.; Hazama, A.; Supplisson, S.; Turk, E.; Wright, E. M., Relaxation kinetics of the Na^+/glucose cotransporter. *Proc. Natl. Acad. Sci. U. S. A.* **1993,** 90, (12), 5767-71.
17. Panayotova-Heiermann, M.; Loo, D. D.; Wright, E. M., Kinetics of steady-state currents and charge movements associated with the rat Na+/glucose cotransporter. *J. Biol. Chem.* **1995,** 270, (45), 27099-105.
18. Mackenzie, B.; Loo, D. D.; Fei, Y.; Liu, W. J.; Ganapathy, V.; Leibach, F. H.; Wright, E. M., Mechanisms of the human intestinal H+-coupled oligopeptide transporter hPEPT1. *J. Biol. Chem.* **1996,** 271, (10), 5430-7.
19. Mackenzie, B.; Loo, D. D.; Panayotova-Heiermann, M.; Wright, E. M., Biophysical characteristics of the pig kidney Na+/glucose cotransporter SGLT2 reveal a common mechanism for SGLT1 and SGLT2. *J. Biol. Chem.* **1996,** 271, (51), 32678-83.
20. Gu, H.; Wall, S. C.; Rudnick, G., Stable expression of biogenic amine transporters reveals differences in inhibitor sensitivity, kinetics, and ion dependence. *J. Biol. Chem.* **1994,** 269, (10), 7124-30.
21. Sucic, S.; Dallinger, S.; Zdrazil, B.; Weissensteiner, R.; Jorgensen, T. N.; Holy, M.; Kudlacek, O.; Seidel, S.; Cha, J. H.; Gether, U.; Newman, A. H.; Ecker, G. F.; Freissmuth, M.; Sitte, H. H., The N terminus of monoamine transporters is a lever required for the action of amphetamines. *J. Biol. Chem.* **2010,** 285, (14), 10924-38.
22. Kaji, D. M., $Na^+/K^+/2Cl^-$ cotransport in medullary thick ascending limb cells: kinetics and bumetanide binding. *Biochim. Biophys. Acta* **1993,** 1152, (2), 289-99.
23. Ovens, M. J.; Davies, A. J.; Wilson, M. C.; Murray, C. M.; Halestrap, A. P., AR-C155858 is a potent inhibitor of monocarboxylate transporters MCT1 and MCT2 that binds to an intracellular site involving transmembrane helices 7-10. *Biochem. J.* **2010,** 425, (3), 523-30.
24. Wright, J. K.; Overath, P., Purification of the lactose:H+ carrier of Escherichia coli and characterization of galactoside binding and transport. *Eur. J. Biochem.* **1984,** 138, (3), 497-508.
25. Wright, J. K.; Riede, I.; Overath, P., Lactose carrier protein of Escherichia coli: interaction with galactosides and protons. *Biochemistry* **1981,** 20, (22), 6404-15.
26. Boorer, K. J.; Frommer, W. B.; Bush, D. R.; Kreman, M.; Loo, D. D.; Wright, E. M., Kinetics and specificity of a H^+/amino acid transporter from Arabidopsis thaliana. *J. Biol. Chem.* **1996,** 271, (4), 2213-20.
